# Nutrient Composition of Germinated Foxtail Millet Flour Treated with Mixed Salt Solution and Slightly Acidic Electrolyzed Water

**DOI:** 10.3390/foods12010075

**Published:** 2022-12-23

**Authors:** Tongjiao Wu, Huiying Li, Jiaxin Li, Jianxiong Hao

**Affiliations:** 1 College of Food Science and Biology, Hebei University of Science and Technology, Shijiazhuang 050018, China; 2 The United Graduate School of Agricultural Sciences, Kagoshima University, 1-21-24 Korimoto, Kagoshima 890-0065, Japan

**Keywords:** germinated millet flour, γ-aminobutyric acid, slightly acidic electrolyzed water, amino acid score

## Abstract

Germination of millet can improve its consumption quality, optimize its nutritional composition, and promote the accumulation of functional components such as γ-aminobutyric acid (GABA). In the present study, foxtail millet was germinated with tap water, a mixed salt solution of 7.5 mmol/L NaCl and 15 mmol/L CaCl_2_, and slightly acidic electrolyzed water (SAEW) with three available chlorine concentrations (ACCs; 10.92, 20.25, and 30.35 mg/L). The effects of the salt solution and SAEW on the germination of foxtail millet and the GABA, crude protein, and amino acid composition of the germinated millet flour were analyzed. The results showed that the salt solution and SAEW treatments promoted the growth of millet sprouts, contributed to the accumulation of GABA in germinated millet flour, and optimized the protein and amino acid composition. The GABA content of germinated foxtail millet flour treated with salt solution for 60 h (336.52 mg/100 g) was 29.5 times higher than that of ungerminated millet flour. In conclusion, the highest GABA content and amino acid scores of germinated millet flour obtained by germination treatment with salt solution at 25 °C and 86% humidity for 60 h were more acceptable for human nutritional requirements.

## 1. Introduction

Foxtail millet (*S. italica*), which is originally from China, is one of the most important agricultural crops in the semi-arid tropics and is considered to be one of the world’s highly productive major cereals [1,2]. Foxtail millet contains high amounts of protein, trace elements, vitamins, and antioxidants [3]. As well as being nutritious, foxtail millet has a lower glycemic index than staple foods made from rice and flour. Some of the polyphenols in it have adjuvant therapeutic effects on diabetes, cancer, and cardiovascular diseases [4]. In recent years, research on millet has attracted the attention of scholars because of its special nutritional composition and physiological functions. Sharma et al. claimed that germination increased the γ-aminobutyric acid (GABA) and polyphenol contents of foxtail millet [5].

Germination is a complex process of physiological and biochemical changes [6,7]; fundamentally, germination changes the nutritional and physicochemical properties of grains [8]. In the field of food science and nutrition, germination is widely used as a major bioprocessing method to improve the nutrient contents of edible seeds, increase protein digestibility, and enrich functional components [9].

Stress causes physiological changes, including reduced transport and uptake of nutrients, and accumulation of chemicals in plant tissues [10]. Koodkaew indicated that 100 mM NaCl treatment increased the flavonoid and ascorbic acid contents in mung bean sprouts [11]. It has been reported that NaCl stress in combination with exogenous Ca^2+^ treatment can induce GABA accumulation in germinating soybeans [12], barley [13], and foxtail millet [3]. Furthermore, it was demonstrated in our previous study that the presence of available chlorine concentration (ACC) in slightly acidic electrolyzed water (SAEW) is also a type of stress that can contribute to the enrichment of GABA in germinated grains [14].

In recent years, SAEW with low ACC of 5–30 mg/L and pH 5.0~6.5 has been shown to have a high anti-infection potential and represents promising prospect in the food industry [15]. SAEW germination could promote the contents of amino acids and phenolic compounds in brown rice and enhance its antioxidant activity [16]. Our previous study found that SAEW promoted the accumulation of GABA in germinated brown millet and effectively reduced its microbial population [2].

Enhancing the nutrient contents of staple grains is an important strategy to overcome deficient consumption of nutrients [17], while germination is an economical and effective method to improve the nutritional quality of grains [5]. Currently, there are numerous studies on methods to promote the germination of grains, mostly focusing on the accumulation of bioactive substances in sprouts. However, little information on the development and utilization of germinated grains themselves and their related products has been reported in the previous studies. Considering the changes in functional substances such as GABA content, proteins, and amino acid composition patterns in germinated grains, it is necessary to analyze and evaluate the nutritional profile of germinated grain flour. In the present study, three germination treatments of millet were used—salt stress, electrolytic water, and tap water treatment (as a control)—and the effects of germination on GABA, crude protein, and amino acid composition in millet flour were compared. The impact of the salt solution and SAEW treatments on the growth of millet sprouts was analyzed. The objective of this study was to compare the influences of the salt solution and SAEW on nutrients during germination of foxtail millet, as well as to determine the optimal conditions for the production of germinated millet flour. Our findings are expected to provide basic data and novel concepts for the processing of millet and the development of germinated millet flour products.

## 2. Materials and Methods

### 2.1. Materials

The cultivar “Yugu 18” of foxtail millet was provided by the Institute of Millet Crops at Hebei Academy of Agriculture and Forestry, China. The dried foxtail millet grains were dehulled in an experimental rubber roll sheller (THU class 35A, Satake rice machine, Tokyo, Japan). The millet was packaged in Ziploc bags and stored at 4 °C for further testing. Standard γ-aminobutyric acid was purchased from Sigma (St. Louis, MO, USA). Analytical-grade chemicals and pure water (Elix Essential UV3, Merck Millipore, Germany) were used in this study.

### 2.2. Preparation of Treatment Solutions

The selection of germination conditions was performed as described in previous reports [2,18]. The foxtail millet was germinated with tap water, a mixed salt solution of 7.5 mmol/L NaCl and 15 mmol/L CaCl_2_, and three kinds of SAEW—denoted as SAEW1 (with 10.92 mg/L ACC), SAEW2 (with 20.25 mg/L ACC), and SAEW3 (with 30.35 mg/L ACC). SAEW was produced using a flow-type electrolysis apparatus (AQUACIDO NDX-250KMS, OSG Company Ltd., Osaka, Japan) with a non-membrane electrolytic cell to electrolyze sodium chloride and hydrochloric acid. After preparation, the SAEW was collected in polypropylene containers and immediately used for subsequent experimental measurements. The pH and ORP of the treatment solutions were measured using a pH meter (Orion Inc., Reston, VA, USA) and an ORP meter (Orion Inc., Reston, VA, USA), respectively. The iodometric method was used to measure the ACC in the treatment solutions using a digital titrator (16900, Hach Company, Loveland, CO, USA). The physicochemical parameters of all treatment solutions are shown in Table 1.

### 2.3. Preparation of Germinated Foxtail Millet and Morphological Measurements of Millet Sprouts

Fifty grams of dehulled millet was rinsed three times with 1 L of the treatment solutions (Table 1). The washed millet was soaked in the treatment solution (1:10, m/v) for 12 h. After soaking, the millet was spread on 4 layers of moist gauze and incubated at a constant temperature of 25 ℃ and 86% humidity, and the treatment solutions were sprayed every 4 h. The germination status of millet at 48 h, 60 h, 72 h, and 84 h was observed, the length of millet sprouts was measured, the number of germinated millet seeds was recorded, and the germination rate (GR) and germination potential (GP) of the millet were calculated according to the following formulae [19]:

(1) $$GR=\frac{NSGn}{TNS}\times 100\%$$

(2) $$GP=\frac{NSG\;\left(3d\right)}{TNS}\times 100\%$$

where GR is the germination rate; NSGn is the number of seeds that germinated within n hours; TNS is the total number of seeds; GP is the germination potential; and NSG (3d) is the number of seeds that germinated within 3 days.

### 2.4. Preparation of Germinated Millet Flour

The 10 g of germinated millet cultured in the different treatment solutions for 48, 60, 72, and 84 h was freeze-dried with a freeze-dryer (LGD-10D, Sihuan Scientific Instrument Factory Co., Ltd., Beijing, China). The freeze-dried sprouted millet was pulverized with a high-speed universal grinder (FSJ302-5, Test Instruments Co., Ltd., Tianjin, China) and sieved through 80 mesh.

### 2.5. GABA Content of Germinated Millet Flour

Two grams of germinated millet flour was mixed with 6 mL of 7% acetic acid, left to stand for 1 h, and then centrifuged at 6000 r/min for 15 min. The supernatant was added to 4 mL of anhydrous ethanol and held at 4 ℃ for 2 h, and then it was centrifuged at 8000 r/min for 20 min. The supernatant was concentrated to dryness by a rotary evaporator (HC-3018, Zhongke Zhongjia Scientific Instrument Co., Ltd. Hefei, Anhui, China), dissolved in 1000 μL of pure water, and then filtered through a 0.45 μm aqueous filter. The GABA content of the germinated foxtail millet flour was determined using an Agilent high-performance liquid chromatograph (HPLC-1260, Agilent Technologies Inc., Santa Clara, CA, USA) with a C_18_ Plus column (250 mm × 4.6 mm, 5 μm) at a column temperature of 40 °C. The mobile phase was sodium acetate (1.36 g of sodium acetate dissolved in 500 mL of ultrapure water, 100 μL of triethylamine, pH adjusted to 7.3 with 50% glacial acetic acid) in phase A and acetonitrile in phase B; A:B = 80:20; detection wavelength: 338 nm; injection volume: 20 μL; flow rate: 0.8 mL/min. The GABA content was calculated using a standard curve (the standard curve is shown in the Supplementary Materials, Figure S1) and the results were expressed as mg/100 g [20].

### 2.6. Determination of Protein

The protein content was assayed according to the method described by Bradford [14], with slight modifications. Briefly, 10 mg of bovine serum albumin (BSA) was dissolved in 100 mL of pure water and kept at 4 °C. Thereafter, 100 mL of Coomassie Brilliant Blue G250 was dissolved in 50 mL of 90% ethanol and 100 mL of 80% phosphoric acid. Finally, pure water was added to adjust the total solution volume to 1000 mL to prepare the G250 solution. Next, 1 mL of BSA and 5 mL of G250 solution were mixed and incubated for 2 min, and a standard curve was plotted by measuring the absorbance at 595 nm (the standard curve is shown in the Supplementary Materials, Figure S2). Furthermore, 0.2 g of germinated millet flour was dissolved in 100 mL of 0.1 mol/L Tris-HCl buffer (pH 8.2) and centrifuged at 1000 r/min for 30 min, and the supernatant was the protein extract. For the measurements, 0.05 mL of protein extract, 0.95 mL of pure water, and 5 mL of G250 solution were mixed and incubated for 2 min. The absorbance was read at 595 nm, and the crude protein content in the germinated millet flour was calculated according to the standard curve.

### 2.7. Analysis of Amino Acid Components

The amino acid components were analyzed according to the method of Monteiro et al., with minor modifications [21]. We weighed 130 mg of germinated millet flour, which was hydrolyzed with 6 mL of 6 mol/L HCl in a sealed reaction kettle at 110 °C for 24 h (no air leakage). After cooling, pure water was added until the total volume of the amino acid extract was 100 mL, and the pH of the extract was adjusted to 2.0 with 6 mol/L NaOH or 6 mol/L HCl. The amino acid extracts were filtered through a 0.45 μm aqueous filter and subsequently placed in an automatic amino acid analyzer for determination, and the results were calculated on a dry basis. Then, the amino acid score was calculated according to the following formula [22]:

(3) $$amino\;acid\;score=\frac{mg\;of\;amino\;acid\;in\;1\;g\;test\;protein}{FAO/WHO\;recommended\;amino\;acid\;pattern}$$

### 2.8. Statistical Analysis

Each treatment was performed in triplicate, and the resulting data were calculated as the mean and the standard deviation. Duncan’s multiple range test (SPSS19.0 for Windows, SPSS Inc., Chicago, IL, USA) was used to analyze the data. Statistical significance was set at a value of *p* < 0.05. The graphs were drawn using Origin 2022 drawing software.

## 3. Results and Discussion

### 3.1. Effect of Treatment Solutions on the Morphological Values of Germinated Millet

**Figure 1:**
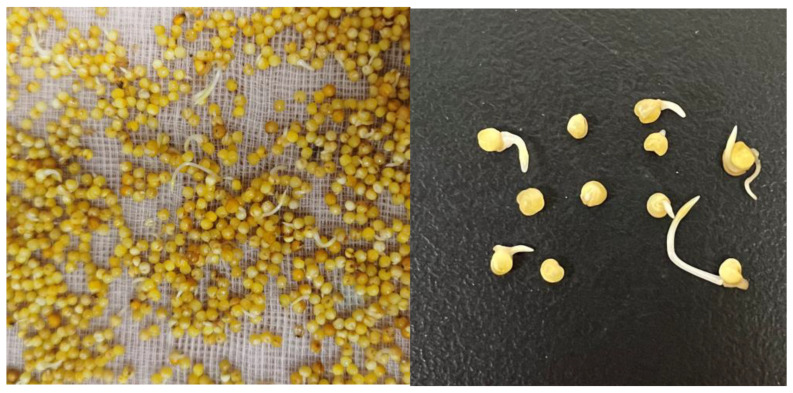
Germinated foxtail millet (germination for 60 h with salt solution).

The effects of the different treatment solutions on the morphological values of the germinated millet were investigated, and the results are shown in Table 2 and Table 3. The appearance of the germinated foxtail millet treated for 60 h with the salt solution is shown in Figure 1 (the photos of the other treatments are shown in the Supplementary Materials, Figure S3). In general, the sprout length and the GR of the germinated millet were significantly changed with the extension of the germination time. SAEW with ACC of 20–30 ppm promoted the growth of millet sprouts, and the length of the sprouts was longer than with the salt solution treatment and the control (Table 2). After germination for 48 h, the length of millet sprouts with the salt solution and SAEW1 treatments was around 0.2 cm, while those treated with SAEW2 and SAEW3 were about 0.38 cm, which was significantly longer than the other treatments (*p* < 0.05). At the late germination (84 h), there were no significant differences in the length of millet sprouts treated with tap water, salt solution, and SAEW1. In contrast, the millet sprout lengths with the SAEW2 and SAEW3 treatments were 2.38 and 2.20 cm, respectively, which were 2.33 and 2.16 times higher than those of the tap water group. Moreover, Liu et al. reported that SAEW with an ACC of approximately 20 ppm promoted the growth of germinated brown rice sprouts [15]. These results show that SAEW can promote the growth of germinated grain sprouts.

Furthermore, the germination capacity and physiological activity of the seeds were estimated by GR and GP, where GR is the number of normally germinated seeds as a percentage of the total number of seeds [19], while GP is the number of normally germinated seeds as a percentage of the total number of seeds at the initial count of seed germination (third day). As shown in Table 3, the GR of millet treated with SAEW was significantly higher than that of both the tap water and salt solution treatments, and the lowest germination rate was found in the control tap water treatment. At 84 h, the highest germination rate of millet was 96.8% in the SAEW1 treatment. Similar results were obtained for GP, where the SAEW treatments showed the highest GP and the salt solution treatment group also had significantly higher GP than the control. Compared with the control, the GP of the SAEW1, SAEW2, and SAEW3 groups improved by 7.80%, 9.93%, and 12.41%, respectively. Compared to the salt solution treatment, the GP of the SAEW groups increased by 5.92%, 8.01%, and 10.45%, respectively. These results suggest that the SAEW had a promotional effect on foxtail millet germination.

### 3.2. Effects of Treatment Solutions on GABA in Germinated Foxtail Millet Flour

**Figure 2:**
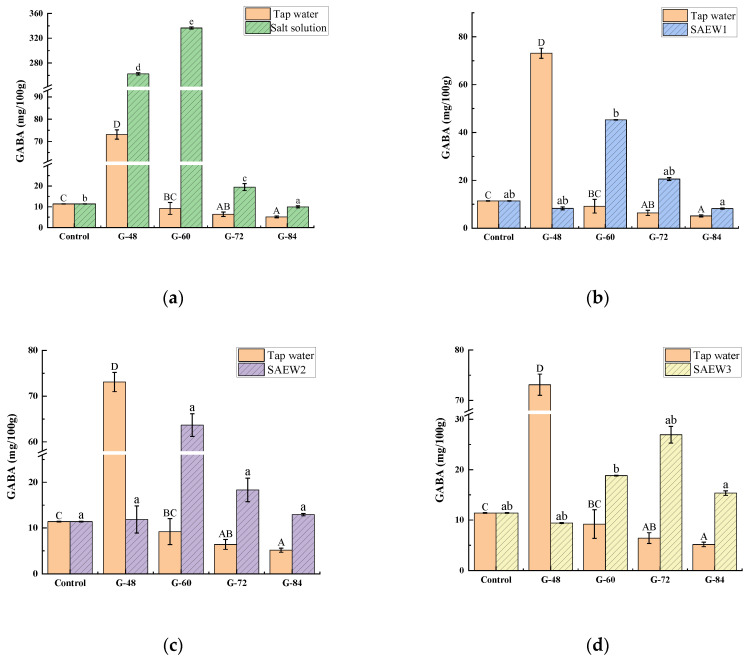
Effects of different germination conditions on the contents of GABA in germinated millet flour: (**a**) salt solution; (**b**) SAEW1; (**c**) SAEW2; (**d**) SAEW3. SAEW, slightly acidic electrolyzed water; SAEW1, 2, and 3 represent SAEW with ACC concentrations of 10.92, 20.25, and 30.35 mg/L, respectively; control, ungerminated millet flour; G-48, 60, 73, and 84 indicate germination for 48, 60, 73, and 84 h, respectively. Different capital letters indicate significant differences for tap water treatment and lowercase letters indicate significant differences for other sample solution treatments (*p* < 0.05). Each value is expressed as the mean ± standard deviation of three replicates.

GABA, a non-protein amino acid with high biological activity, is one of the major neurotransmitters in the brain. GABA regulates blood pressure, heart rate, perceptions of pain and anxiety, serum lipid levels, and insulin secretion, and it has pharmacological effects such as antihypertensive activity, sedative effects, inhibition of sympathetic neurotransmitters, and prevention of diabetes [17,20]. As an endogenous phytochemical, low contents of GABA can be found in many plants; however, germinated grains contain more GABA than ungerminated ones [2,23]. Therefore, the effects of different germination conditions on the contents of GABA in germinated foxtail millet flour were evaluated, and the results are shown in Figure 2.

The initial GABA content of the ungerminated millet flour was 11.4 mg/100 g. The GABA contents in germinated foxtail millet flour under all treatment conditions showed an increasing tendency at early germination and then gradually decreased. During the germination process, proteins are hydrolyzed by proteases to produce large amounts of glutamate. Moreover, germination can activate glutamic acid decarboxylase (GAD) to catalyze the decarboxylation of glutamate to produce GABA. Therefore, the GABA content in millet seeds showed an increasing tendency at early germination. With the continuous accumulation of GABA, the activity of γ-aminobutyric acid transaminase is stimulated to catalyze the reaction of GABA with α-ketoglutarate to form glutamate and succinate semialdehyde. At this time, the rate of GABA production is much less than the rate of GABA consumption, resulting in a decrease in GABA content [5,23,24].

After 48 h of germination with the tap water treatment, the GABA content of the millet flour reached the highest level (73.10 mg/100 g), which was 6.4 times higher than that of ungerminated millet. This result is consistent with the findings of previous studies showing that germination could increase the GABA content in grains [5,25]. For the SAEW treatments (Figure 2b–d), the GABA content reached a maximum at 60 h for SAEW1 and SAEW2, and at 72 h for SAEW3, with 45.25 mg/100 g, 63.38 mg/100 g, and 26.93 mg/100 g, respectively, all of which were significantly higher than the GABA content of ungerminated millet (*p* < 0.05). GABA accumulated rapidly in response to variety of stresses, such as acidosis, anoxia, cold, heat, drought, and salt [13]. ACC and other oxidizing substances such as hydroxyl radicals exist in SAEW, which also could impose a kind of stress, causing the accumulation of GABA [20]. In addition, the GABA content in the germinated millet flour reached a peak of 336.52 mg/100 g after 60 h of germination with the salt solution treatment, which was 29.5 times higher than that of ungerminated millet (Figure 2a). NaCl stress, as an adverse stress germination mode, stimulates enzyme activity and increases GAD activity, while the presence of Ca^2+^ similarly increases GAD activity. The synergistic effect of NaCl and Ca^2+^ greatly increases GAD activity, which further promotes the decarboxylation of glutamate to produce GABA, resulting in a much higher GABA content in millet treated with salt solution than with other germination methods [3].

### 3.3. Effects of Treatment Solutions on Protein in Germinated Foxtail Millet Flour

**Figure 3:**
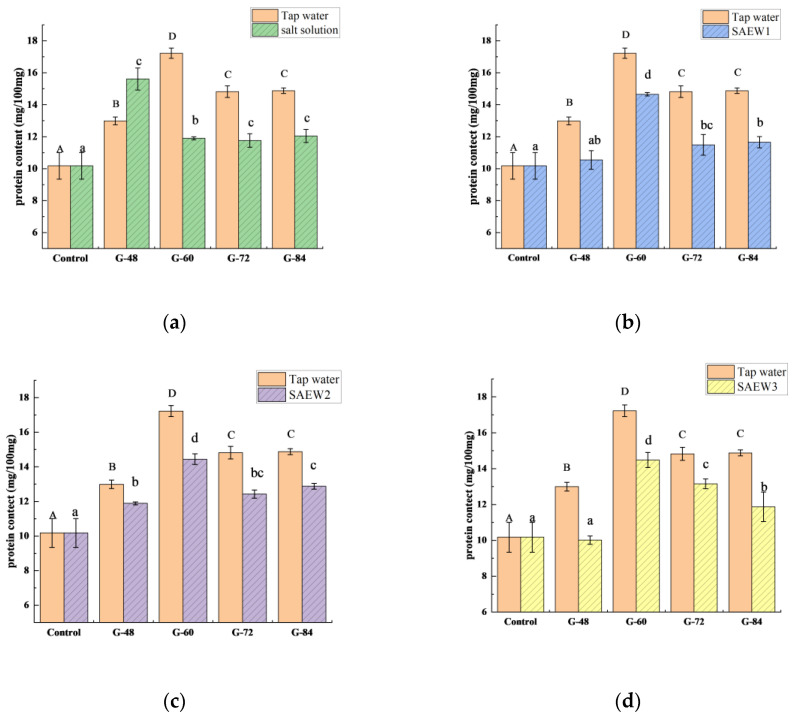
Effects of different germination conditions on the protein contents in germinated millet flour: (**a**) salt solution; (**b**) SAEW1; (**c**) SAEW2; (**d**) SAEW3. SAEW, slightly acidic electrolyzed water; SAEW1, 2, and 3 represent SAEW with ACC concentrations of 10.92, 20.25, and 30.35 mg/L, respectively; control, ungerminated millet flour; G-48, 60, 73, and 84 indicate germination for 48, 60, 73, and 84 h, respectively. Different capital letters indicate significant differences for tap water treatment and lowercase letters indicate significant differences for other sample solution treatments (*p* < 0.05). Each value is expressed as the mean ± standard deviation of three replicates.

Germination also has an effect on protein levels. The effects of different germination conditions on the protein contents in the germinated foxtail millet flour are shown in Figure 3. Generally, the protein content of the germinated foxtail millet flour showed a tendency to increase and then decrease with the extension of the germination time. The salt solution treatment showed an increasing trend in protein content from 0 to 48 h. After germination for 48 h, the protein content was 15.61 mg/100 mg, which was significantly higher than that of the tap water treatment (Figure 3a). The SAEW and tap water treatments showed an increase in protein content from 0 to 60 h (Figure 3b–d). Among them, the highest protein content was 17.23 mg/100 mg at 60 h of germination with the tap water treatment. In the initial germination period, the increase in protein content is due to the formation of essential amino acids and can also be increased by the activation of germination enzymes [26]. However, the protein content in the period of germination is dependent on the balance between the degradation and biosynthesis of proteins [27]. During germination, proteins are degraded by enzymes, converted into transportable amides, and supplied to the growing parts of the seedlings. When protein degradation is greater than biosynthesis, the protein content shows a decreasing trend.

Throughout the germination process, except for the salt solution treatment for 48 h, the protein content of the germinated millet flour in all treatments was significantly less than that of the tap water treatment. It is certain that the crude protein content of germinated millet flour in each treatment group was significantly higher than that of the ungerminated millet flour. However, the protein quality of food depends on the composition and availability of amino acids [28]. Therefore, it is necessary to further analyze the contents and composition of amino acids in germinated millet flour.

### 3.4. Free Amino Acids in Germinated Foxtail Millet Flour with Different Treatment Solutions

Since the highest GABA contents in the germinated foxtail millet flour were found after 60 h of salt solution treatment, 60 h of SAEW2 treatment, and 48 h of tap water treatment, germinated millet with these three treatment conditions—as well as ungerminated millet as a control—was selected for amino acid composition analysis. Germination stimulates enzymatic activity, and the proteins in millet seeds are continuously decomposed into the amino acids and peptides required for germination [27,29]. As shown in Table 4, the amino acid species of the germinated millet flour were consistent with those of the ungerminated millet flour, but the contents of each amino acid changed significantly (*p* < 0.05) after germination. Germinated millet flour contained 17 amino acids, including 8 essential amino acids, and the contents of various amino acids were significantly higher compared with ungerminated millet flour (*p* < 0.05). In germinated millet flour, the highest glutamic acid content was found, followed by leucine, alanine, aspartic acid, proline, valine, and phenylalanine in decreasing order. For instance, the leucine content of germinated millet flour varied significantly between treatments (*p* < 0.05), with the highest content in the SAEW treatment, followed by the tap water treatment, the salt solution treatment, and the ungerminated millet flour. Shen et al. reported that germination improved the contents of most free amino acids in brown rice [30]. Tyagi et al. claimed that SAEW promoted the accumulation of amino acid content in germinating brown rice. The composition of amino acids might be significant to determine the health and nutritional role of brown rice [16].

The nutritional value of protein in a food should be evaluated from both “quantity” and “quality” perspectives, including the contents and patterns of essential amino acids [28]. Since there are certain types and ratios of amino acids required by humans, the nutritional value of the protein is examined depending on whether the contents and ratio of essential amino acids in the protein meet the demand. Within a safe protein intake level, the amino acid score indicates whether the absorbed dietary nitroglycerin is effective in meeting the indispensable amino acid requirement. This is defined as the ratio of the content of an essential amino acid per gram of test protein to the corresponding essential amino acid content in the FAO/WHO-recommended pattern [22]. The essential amino acid composition pattern and AAS in germinated millet flour protein are shown in Table 5. According to the FAO/WHO-recommended pattern, the essential amino acid/total amino acid ratio (EAA/TAA) of all germinated millet flour treatments was greater than the FAO/WHO-recommended value (0.4), and the essential amino acid/non-essential amino acid ratio (EAA/NAA) of all treatments was greater than 0.6. Moreover, the highest EAA/TAA and EAA/NAA were found in the germinated millet flour treated with the salt solution, followed by the SAEW treatment. In addition, the AAS values of each essential amino acid were significantly higher (*p* < 0.05) in the salt solution treatment than in the other treatments. These results indicate that germination can significantly enhance the nutritional value of protein in millet seeds. Although the free amino acid content was significantly higher in the SAEW treatment than in the salt solution treatment (Table 4), the latter had higher AAS values. It can be concluded that the salt solution germination treatment can promote the nutritional value of foxtail millet.

## 4. Conclusions

The effects of the salt solution and SAEW treatments on the germination of foxtail millet and the nutritional composition in germinated millet flour were compared in this study. The germination length of millet sprouts in the SAEW2 and SAEW3 treatments was significantly longer than in the other treatments in the same period. The germination rate and germination potential of the salt solution treatment were significantly higher than those of the tap water treatment. The GABA content in the germinated millet flour showed a tendency to increase and then decrease with the prolongation of the germination time. The highest GABA content of the germinated millet flour was found at 60 h for the SAEW2 and salt solution treatments and at 48 h for the tap water treatment. The protein and amino acid contents were significantly higher in all germinated millet flours than in the ungerminated group during germination. The amino acid pattern of the germinated millet flour treated with the salt solution was more favorable than that of the other treatments and had the highest nutritional value. In conclusion, the GABA content of the germinated foxtail millet flour treated with the salt solution for 60 h (336.52 mg/100 g) was 29.5 times higher than that of the ungerminated millet flour and had a high-quality amino acid pattern. Therefore, the optimal conditions for processing germinated millet flour were determined to be 25 °C temperature, 86% humidity, and 60 h germination treatment with the salt solution.

## Figures and Tables

**Table 1:** The physicochemical parameters of the treatment solutions ^1^.

Treatment Solutions	pH	ORP (mV)	ACC (mg/L)
Tap water	7.51 ± 0.03	308 ± 4	-
SAEW1	5.73 ± 0.04	834 ± 7	10.92 ± 0.27
SAEW2	5.85 ± 0.05	855 ± 13	20.25 ± 0.14
SAEW3	5.95 ± 0.01	861 ± 6	30.35 ± 0.36
Salt solution	7.26 ± 0.02	317 ± 8	-

^1^ Tap water was used as a control. SAEW, slightly acidic electrolyzed water; ACC, available chlorine concentration; ORP, oxidation–reduction potential; “-”, not detected. Each value is expressed as the mean ± standard deviation of three replicates.

**Table 2:** Effects of different germination treatments on the length of foxtail millet sprouts ^1^.

Treatment Solutions	Length of Millet Sprouts (cm)			
	48 h	60 h	72 h	84 h
Tap water	0.279 ± 0.039 bA	0.523 ± 0.052 aB	0.557 ± 0.096 aB	1.020 ± 0.098 aC
Salt solution	0.200 ± 0.019 aA	0.481 ± 0.028 aB	0.753 ± 0.118 abC	1.137 ± 0.109 aD
SAEW1	0.191 ± 0.041 aA	0.673 ± 0.131 bB	0.914 ± 0.132 bC	1.160 ± 0.292 aD
SAEW2	0.376 ± 0.071 cA	0.815 ± 0.121 cB	1.654 ± 0.374 cC	2.379 ± 0.103 bD
SAEW3	0.382 ± 0.058 cA	0.799 ± 0.054 cB	1.626 ± 0.319 cC	2.201 ± 0.093 bD

^1^ SAEW, slightly acidic electrolyzed water; SAEW1, 2, and 3 represent SAEW with ACC concentrations of 10.92, 20.25, and 30.35 mg/L, respectively; tap water was used as a control. Different lowercase letters in each column indicate significant differences (*p* < 0.05). Different capital letters in each row indicate significant differences (*p* < 0.05). Each value is expressed as the mean ± standard deviation of three replicates.

**Table 3:** Effects of different germination treatments on the germination rate and germination potential of foxtail millet ^1^.

Treatment Solutions	GR (%)			
	48 h	60 h	72 h (GP %)	84 h
Tap water	40.0 ± 0.82 aA	46.3 ± 0.96 aB	70.5 ± 2.08 aC	92.5 ± 0.58 aD
Salt solution	42.8 ± 0.96 bA	48.0 ± 0.82 aB	71.8 ± 2.06 bC	93.8 ± 0.96 bD
SAEW1	48.8 ± 0.96 cA	52.5 ± 1.73 bB	76.0 ± 1.73 cC	96.8 ± 0.96 dD
SAEW2	48.7 ± 1.15 cA	55.0 ± 2.45 bcB	77.5 ± 2.65 cC	94.8 ± 0.96 bcD
SAEW3	48.5 ± 0.58 cA	57.8 ± 3.69 cB	79.3 ± 1.89 cC	95.5 ± 0.58 cD

^1^ GR, germination rate; GP, germination potential, i.e., the GR on the third day (72 h) of germination; SAEW, slightly acidic electrolyzed water; SAEW1, 2, and 3 represent SAEW with ACC concentrations of 10.92, 20.25, and 30.35 mg/L, respectively; tap water was used as a control. Different lowercase letters in each column indicate significant differences (*p* < 0.05). Different capital letters in each row indicate significant differences (*p* < 0.05). Each value is expressed as the mean ± standard deviation of three replicates.

**Table 4:** Amino acid profiles of different germinated foxtail millet treaments ^1^.

Amino Acid (g/100 g)	Tap Water-48 h	Salt Solution-60 h	SAEW2-60 h	Ungerminated
Aspartic acid	0.813 ± 0.0003 c	0.785 ± 0.0000 b	0.807 ± 0.0003 c	0.617 ± 0.0005 a
Threonine *	0.437 ± 0.0002 c	0.427 ± 0.0002 b	0.436 ± 0.0003 c	0.352 ± 0.0001 a
Serine	0.512 ± 0.0002 c	0.502 ± 0.0003 b	0.510 ± 0.0002 c	0.426 ± 0.0000 a
Glutamate	2.160 ± 0.0002 c	2.114 ± 0.0001 b	2.158 ± 0.0005 c	1.864 ± 0.0001 a
Glycine	0.327 ± 0.0002 c	0.317 ± 0.0003 b	0.325 ± 0.0002 c	0.242 ± 0.0003 a
Alanine	0.973 ± 0.0003 b	0.980 ± 0.0003 c	1.004 ± 0.0002 d	0.811 ± 0.0009 a
Cystine	0.033 ± 0.0001 b	0.035 ± 0.0005 b	0.033 ± 0.0003 b	0.021 ± 0.0000 a
Valine *	0.615 ± 0.0004 c	0.609 ± 0.0004 b	0.623 ± 0.0005 c	0.482 ± 0.0002 a
Methionine *	0.056 ± 0.0003 b	0.109 ± 0.0002 d	0.102 ± 0.0001 c	0.023 ± 0.0003 a
Isoleucine *	0.444 ± 0.0004 b	0.438 ± 0.0003 b	0.450 ± 0.0001 c	0.353 ± 0.0001 a
Leucine *	1.505 ± 0.0005 c	1.499 ± 0.0003 b	1.526 ± 0.0003 d	1.253 ± 0.0004 a
Tyrosine	0.240 ± 0.0003 b	0.238 ± 0.0005 b	0.242 ± 0.0004 b	0.100 ± 0.0006 a
Phenylalanine *	0.601 ± 0.0004 c	0.592 ± 0.0003 b	0.609 ± 0.0004 c	0.497 ± 0.0012 a
Lysine *	0.257 ± 0.0004 c	0.250 ± 0.0003 b	0.257 ± 0.0004 c	0.175 ± 0.0001 a
Histidine *	0.229 ± 0.0002 c	0.221 ± 0.0003 b	0.228 ± 0.0003 c	0.178 ± 0.0010 a
Arginine	0.390 ± 0.0004 c	0.357 ± 0.0004 b	0.364 ± 0.0001 c	0.288 ± 0.0010 a
Proline	0.732 ± 0.0005 b	0.727 ± 0.0002 b	0.744 ± 0.0004 c	0.701 ± 0.0010 a

^1^ Tap water-48 h, Salt solution-60 h, and SAEW2-60 h represent the germinated millet flour treated with tap water for 48 h, salt solution for 60 h, and slightly acidic electrolyzed water with an ACC concentration of 20.25 mg/L for 60 h, respectively; *, essential amino acids. The different letters indicate significant differences (*p* < 0.05). Each value is expressed as the mean ± standard deviation of three replicates.

**Table 5:** Composition patterns and AAS of essential amino acids in foxtail millet protein ^1^.

Amino Acid	Amino Acid Score (AAS)				FAO/WHO-Recommended Amino Acid Pattern
	Tap Water-48 h	Salt Solution-60 h	SAEW2-60 h	Ungerminated	
Threonine	84.02	102.55	75.52	86.35	40
Valine	94.74	116.85	86.31	94.74	50
Methionine + cystine	19.56	39.50	26.82	12.36	35
Isoleucine	85.52	105.07	77.91	86.75	40
Leucine	165.53	205.56	150.97	175.89	70
Phenylalanine + tyrosine	107.80	132.73	98.24	97.77	60
Lysine	35.94	43.65	32.30	31.31	55
EAA/TAA	0.401	0.406	0.406	0.395	
EAA/NAA	0.671	0.684	0.684	0.654	

^1^ Tap water-48 h, Salt solution-60 h, and SAEW2-60 h represent the germinated millet flour treated with tap water for 48 h, salt solution for 60 h, and slightly acidic electrolyzed water with an ACC concentration of 20.25 mg/L for 60 h, respectively. EAA, essential amino acid content; TAA, total amino acid content, NAA: non-essential amino acid content.

## Data Availability

The data are available from the corresponding author.

## Supplementary Materials

The following supporting information can be downloaded at: https://www.mdpi.com/article/10.3390/foods12010075/s1, Figure S1: GABA standard curve; Figure S2: Protein standard curve; Figure S3: Germinated foxtail millet with different treatments for 60 h.

## Abbreviations

GABA	γ-Aminobutyric acid
SAEW	Slightly acidic electrolyzed water
ACC	Available chlorine concentration
GR	Germination rate
GP	Germination potential
BSA	Bovine serum albumin
GAD	Glutamic acid decarboxylase
