# Supplementary material for: Nutrient Composition of Germinated Foxtail Millet Flour Treated with Mixed Salt Solution and Slightly Acidic Electrolyzed Water
Source: Foods. 2022 Dec 23;12(1):75. doi: 10.3390/foods12010075 (PMC9818339; doi:10.3390/foods12010075)
Supplement: Supplementary file 1 [file foods-12-00075-s001.zip › foods-2054784-SI.pdf]

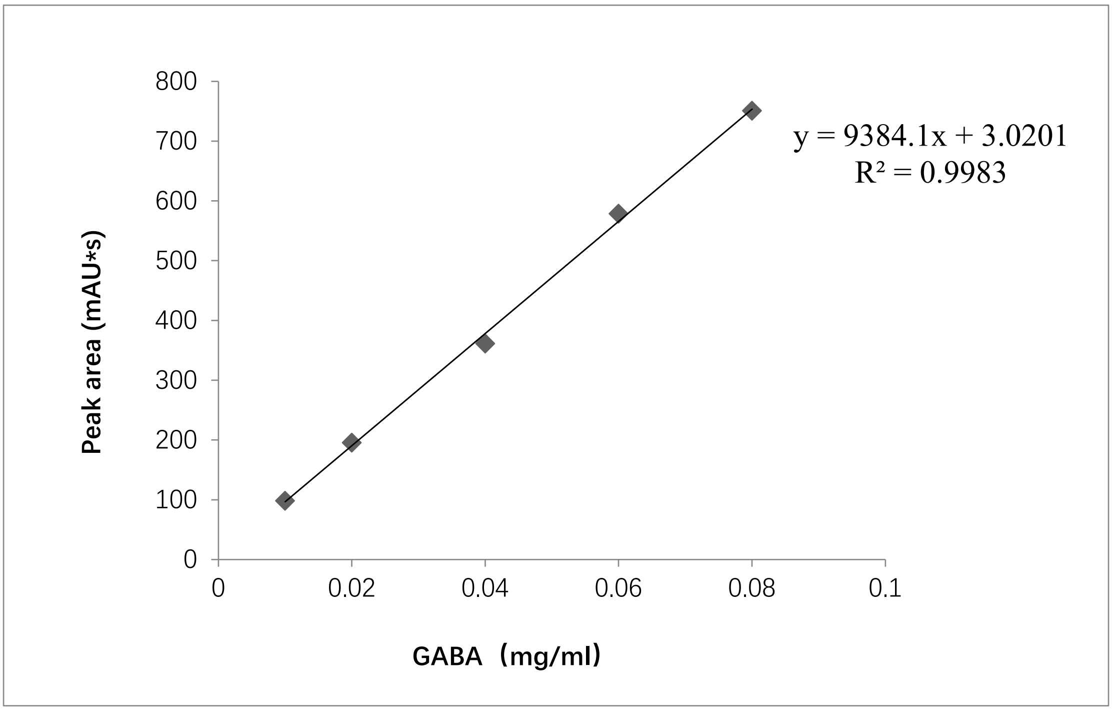

Figure S1. GABA standard curve.

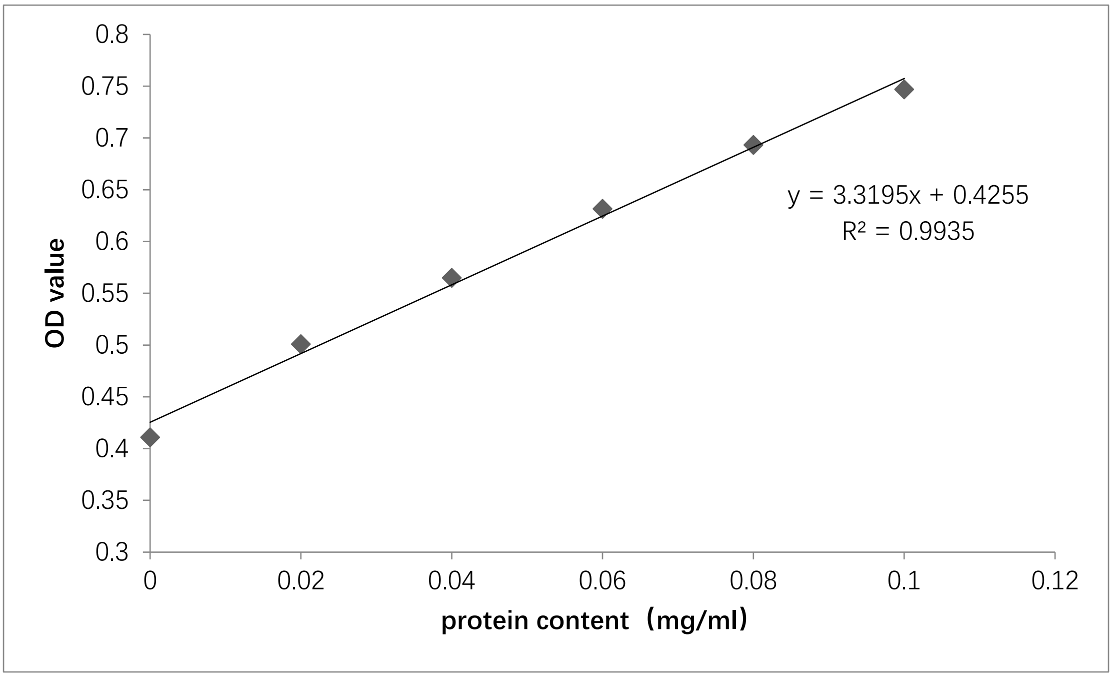

Figure S2. Protein standard curve

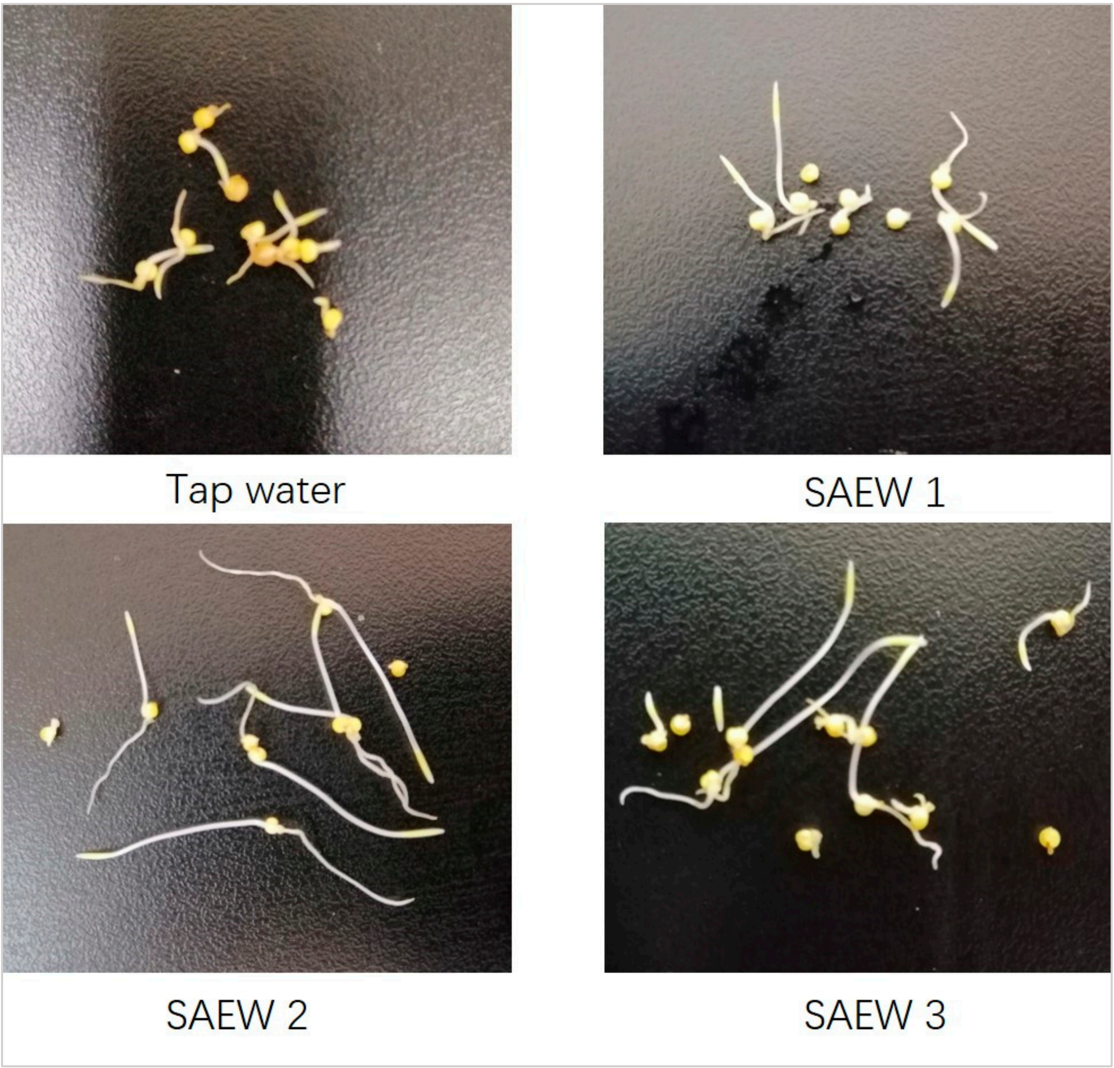

Figure S3. Germinated foxtail millet with different treatments for 60h.
